# Resource-Efficient Multicast URLLC Service in 5G Systems

**DOI:** 10.3390/s24082536

**Published:** 2024-04-15

**Authors:** Artem Krasilov, Irina Lebedeva, Ruslan Yusupov, Evgeny Khorov

**Affiliations:** 1 Telecommunication Systems Lab, HSE University, Moscow 101000, Russia; krasilov@iitp.ru (A.K.);; 2 Wireless Networks Lab, Institute for Information Transmission Problems of the Russian Academy of Sciences, Moscow 127051, Russia

**Keywords:** 5G, multicast, URLLC, massive MIMO, MCS

## Abstract

Many emerging applications, such as factory automation, electric power distribution, and intelligent transportation systems, require multicast Ultra-Reliable Low-Latency Communications (mURLLC). Since 3GPP Release 17, 5G systems natively support multicast functionality, including multicast Hybrid Automatic Repeat Request and various feedback schemes. Although these features can be promising for mURLLC, the specifications and existing studies fall short in offering guidance on their efficient usage. This paper presents the first comprehensive system-level evaluation of mURLLC, leveraging insights from 3GPP specifications. It points out (i) how mURLLC differs from traditional multicast broadband wireless communications, and (ii) which approaches to provide mURLLC require changing the paradigm compared with the existing solutions. Finally, the paper provides recommendations on how to satisfy strict mURLLC requirements efficiently, i.e., with low channel resource consumption, which increases the capacity of 5G systems for mURLLC. Simulation results show that proper configuration of multicast mechanisms and the corresponding algorithms for mURLLC traffic can reduce resource consumption up to three times compared to the baseline solutions proposed for broadband multicast traffic, which significantly increases the system capacity.

## 1. Introduction

Ultra-Reliable Low-Latency Communications (URLLC) is a new type of service supported in 5G systems. While URLLC Quality of Service (QoS) requirements on latency and reliability depend on the application, the typical values considered by 3GPP are 1–10 ms for latency and $1\times 10^{-4}$–$1\times 10^{-9}$ for reliability [1]. In Releases 15/16, 3GPP has developed a New Radio (NR) access technology that enables unicast URLLC service, i.e., the delivery of a data stream to/from a single User Equipment (UE). For that, NR supports mini-slots, new robust Modulation and Coding Schemes (MCSs), fast Hybrid Automatic Repeat Request (HARQ), etc.

Many emerging applications, such as factory automation, electric power distribution, and intelligent transportation systems, require the support of *multicast URLLC (mURLLC)*, i.e., the delivery of the same data from a base station (called gNB) to a group of UEs with strict requirements on latency and reliability. The straightforward approach to enable multicast is to convert a multicast stream into multiple unicast streams addressed to each UE, which manifold increases the channel resource consumption. Being inefficient for a low number of UEs, it becomes completely unsuitable for massive mURLLC because it increases delays above those required for the UEs served last. To save channel resources and reduce delays, since Release 17, NR supports new mechanisms that enable native multicast for different traffic types, e.g., voice and IPTV. Release 18 only slightly enhances multicast functionality, e.g., by enabling data reception in inactive state and dynamic switching between multicast/unicast transmission [2], while Release 17 adds new multicast mechanisms to the NR protocol stack [3,4,5,6] and system architecture [7]. The detailed description and analysis of the novelties can be found in recent papers [8,9,10,11], which mainly focus on multicast broadband traffic. In contrast, this paper focuses on those mechanisms and algorithms that are needed for mURLLC.

The first mechanism that improves reliability is *multicast HARQ*. If a multicast packet has not been delivered to some UEs, the gNB can schedule a HARQ retransmission that can be addressed either to the original multicast UE group or to a particular UE. HARQ retransmissions can be carried out either based on the feedback from UE(s) or blindly. Note that 3GPP specifications do not define how to select the number of HARQ retransmissions and their parameters (e.g., MCS).

The second mechanism enables several ways in which UEs can provide feedback about the decoding status (success or failure) of previous transmissions. This feedback is needed to perform conditional HARQ retransmissions, i.e., to decide on a transmission retry based on the set of the UEs to which the data have not been delivered yet. However, the 3GPP specifications do not describe how to configure such feedback.

In addition to these two mechanisms, in this paper, we study various transmission parameters selection algorithms, which are left for implementation by vendors. Specifically, since Massive Multiple Input Multiple Output (M-MIMO) is a key feature of 5G systems, the gNB shall implement algorithms that select a precoder, allocate frequency resources, and select a single MCS for each multicast transmission. Without proper configuration of the multicast mechanisms and the above-mentioned transmission parameters, either the strict reliability and latency requirements may be not satisfied or the channel resource consumption is too high, which limits the cell capacity.

Despite being important, the area of mURLLC has not been well addressed in the literature. Many works evaluate the performance of new multicast mechanisms [12,13,14,15] and propose new transmission parameters selection algorithms [16,17,18,19,20]. However, these works only consider multicast broadband traffic (e.g., file transfer, IPTV) with moderate latency and reliability requirements, while mURLLC imposes much stricter requirements. Thus, the considered multicast solutions may not be suitable for mURLLC. In other work, many URLLC-aware transmission parameters selection [21,22,23,24] and scheduling [25,26,27,28] algorithms have been designed for unicast traffic. An open question is how to adapt them for the multicast case. The paper aims to fill this research gap. It shows which approaches to provide mURLLC require changing the paradigm and which approaches can be inherited from existing ones. Table 1 summarizes these findings, which are discussed in detail in the following sections. An arrow with the label “new” means that the paper proposes a modification of a solution to mURLLC. An arrow without the label “new” means that the solution or a specific subset of solutions that is defined in the paper can be applied to mURLLC. The sign “X” means that the solutions proposed for other areas are inefficient for mURLLC.

While analyzing the algorithms, we pay much attention to the computational complexity because mURLLC requires data delivery to multiple UEs and the decisions needs to be made within a short time. Thus, only a limited set of algorithms with low complexity can be applied for mURLLC.

The contributions of the paper are as follows:

1. We review various existing transmission parameter selection algorithms developed for multicast broadband traffic and unicast URLLC traffic and determine how to adapt them for mURLLC;
2. We carry out extensive performance evaluation and comparison of various algorithms under the same conditions using link-level and system-level simulations;
3. Based on extensive simulation results, we provide a set of recommendations on how to configure the new multicast mechanisms and determine the algorithms providing mURLLC with low resource consumption, which, in turn, increases system capacity.

Note that some recommendations contradict those for multicast broadband traffic and unicast URLLC traffic. For example, several works [29,30] report that feedback-based multicast HARQ retransmissions are inefficient for broadband traffic because they slightly reduce the downlink resource consumption while significantly increasing the uplink resource consumption. In contrast, we show that some feedback schemes do significantly reduce the overall resource consumption under strict reliability constraint. Another example of a non-trivial recommendation is related to the resource allocation algorithm. In contrast to the existing works [25,26] that recommend using the Frequency-Selective (FS) scheduler (i.e., allocate resource blocks taking into account their quality) for unicast URLLC traffic, we show that the gain of an FS scheduler for a large multicast group is below 5% with respect to a non-FS scheduler while the complexity increases by 40%. Thus, the type of scheduler (FS or non-FS) should be selected depending on the multicast group size.

The rest of the paper is organized as follows. We describe the considered scenario and formulate the problem in Section 2. In Section 3, we analyze various transmission parameter selection algorithms for multicast traffic and show how to adapt them to mURLLC. We evaluate their performance in Section 4. Section 5 concludes the paper with recommendations on providing resource-efficient mURLLC.

## 2. System Model and Problem Statement

**Figure 1:**
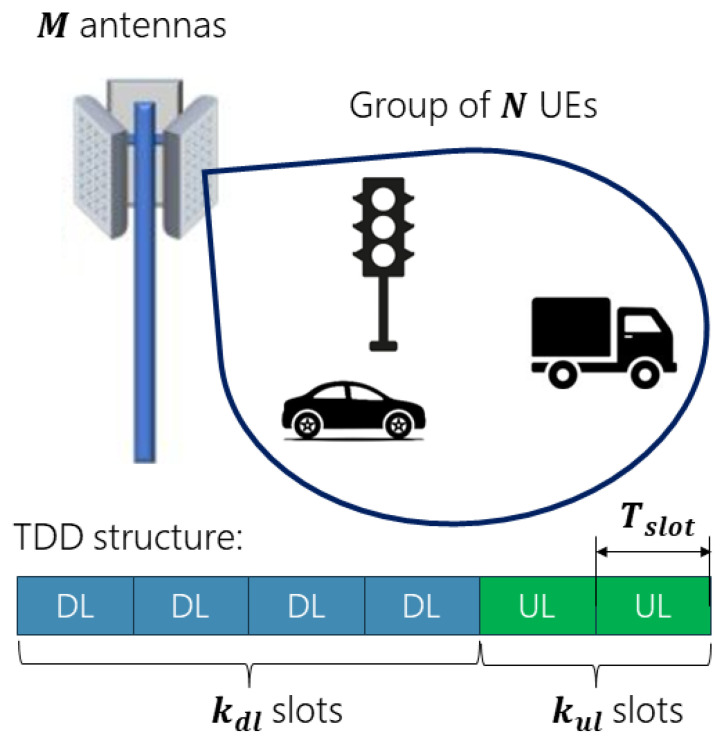
System model illustration.

Consider a gNB providing the multicast URLLC service for *N* UEs (see Figure 1). We suppose that all UEs are connected to the gNB. Thus, the gNB allocates uplink resources for transmission of uplink control information (e.g., UE feedback) and Sounding Reference Signals (SRSs) (tspecifications introduce both multicast and broadcast modes. In contrast to multicast mode, broadcast mode allows transmitting to UEs not connected to the gNB. In broadcast mode, UEs do not send any feedback, and the gNB cannot guarantee strict URLLC requirements [8]). The QoS requirements of an mURLLC stream are as follows: (i) the latency for each packet (i.e., the time interval between packet arrival at the gNB and its delivery to all UEs in the multicast group) shall not exceed $D^{QoS}$ and (ii) the Packet Loss Ratio (PLR) shall be lower than $PLR^{QoS}$. A multicast packet is considered lost if it is not delivered within a given latency budget to at least one UE. The typical values of $D^{QoS}$ and reliability (i.e., $1-PLR^{QoS}$) for mURLLC traffic are provided at the beginning of Section 1.

We consider an M-MIMO system: the gNB is equipped with a large number *M* of antennas. To simplify the description, we consider single antenna UEs. However, the results can be easily extended for multi-antenna UEs. To provide efficient M-MIMO operation, the gNB uses a Time Division Duplex (TDD) scheme with a periodic structure of downlink (DL) and uplink (UL) time slots, which have equal duration $T_{slot}$. Specifically, $k_{dl}$ DL slots used for data transmission are followed by $k_{ul}$ UL slots used for UE feedback and/or Sounding Reference Signals (SRSs). SRSs are transmitted by UEs with the period $T_{SRS}$. Thanks to channel reciprocity in the case of TDD, the gNB can use SRSs to estimate both DL and UL channel quality. In a frequency domain, each slot is divided into *B* Resource Blocks (RBs), where *B* depends on the bandwidth and the used numerology.

For each multicast stream, the gNB solves the following problems, see Table 2. First, in the long-term timescale, the gNB configures the maximum number of transmission attempts (including HARQ retransmissions) and selects the feedback scheme and the sounding period $T_{SRS}$. Second, in the short-term timescale, i.e., for each transmission attempt, the gNB dynamically: (i) constructs a precoder in each RB, (ii) allocates RBs taking into account their quality and the current buffer size, and (iii) selects an MCS. Since the 3GPP does not describe how to address these problems, in the following sections, we consider various solutions and evaluate which of them can provide resource-efficient mURLLC service, i.e., provide low overall (DL + UL) channel resource consumption while satisfying strict mURLLC requirements. Low channel resource consumption allows an increased system capacity, i.e., increasing the number of concurrent flows with satisfied QoS requirements and/or increasing the load of each flow.

## 3. Analyses of Possible Solutions and Their Adaptation to mURLLC

In this section, we consider solutions aimed to address the problems identified in Section 2. As detailed in Section 1, due to the lack of algorithms/solutions specifically developed for mURLLC, in this paper, we adapt the solutions proposed either for broadband multicast traffic or for unicast URLLC traffic. In the latter case, we show how to extend/modify solutions such that they can work with multiple receivers.

The following sections are structured as follows. First, we analyze how the gNB can select long-term transmission parameters: the maximum number of transmission attempts, the feedback scheme, and the sounding period. Then, we consider how the gNB selects parameters for each particular transmission: the precoder, RBs, and the MCS.

### 3.1. The Maximum Number of Transmission Attempts

The number of transmission attempts (TXs) affordable for each multicast packet depends on the latency limitation and the TDD configuration. In particular, for very strict latency requirements, i.e., $D^{QoS}<\left(k_{dl}+k_{ul}\right)T_{slot}$, HARQ retransmissions cannot be delivered in time. Thus, only a single robust TX is possible. For moderate latency requirements, i.e., $D^{QoS}∼\left(k_{dl}+k_{ul}\right)T_{slot}$, the gNB can obtain UE feedback and make a conditional HARQ retransmission if the initial TX fails. Taking into account typical values of $D^{QoS}∼1–10$ ms for URLLC and $T_{slot}∼0.5–1$ ms for numerologies used in frequency bands below 6 GHz, in the paper, we focus on two cases: (i) One TX case when a single TX is possible, and (ii) Two TXs case, i.e., one initial TX and one HARQ retransmission are possible. We assume that the gNB selects this parameter at the beginning of the multicast flow and changes it very rarely (e.g., when the set of served UEs or their channel conditions significantly change).

### 3.2. The Feedback Scheme

For each multicast stream, the gNB can configure one out of three feedback schemes. With the first scheme, which, hereafter, we call No feedback, the gNB does not allocate uplink resources for the feedback transmission. Thus, conditional HARQ cannot be used with this scheme. In contrast, with the second scheme, called *ACK/NACK feedback*, the gNB allocates a separate uplink resource for each UE such that the UE can send a positive (ACK) or negative (NACK) acknowledgment for each transmission. With the third scheme, called *NACK-only feedback*, the gNB configures a single uplink resource where only the UEs that have failed to decode the transmission send NACK. Thus, with this scheme, the gNB schedules an HARQ retransmission but does not know which UEs require it, which complicates transmission parameters selection for subsequent TXs.

For each multicast stream, the gNB can select a feedback scheme depending on the stream QoS requirements and the number of available TXs. Specifically, for the one TX case (i.e., for strict latency requirements), the gNB can use either No feedback or NACK-only feedback schemes. In the case of the No feedback scheme, to provide high reliability, the gNB shall select a very robust MCS, which leads to huge resource consumption. In contrast, the usage of the NACK-only feedback scheme allows selecting proper MCS by taking into account UE feedback at the cost of moderate uplink overhead. For two or more TXs, the gNB can use either the NACK-only or ACK/NACK feedback schemes. Comparing these schemes, the ACK/NACK feedback scheme allows reducing resource consumption for HARQ retransmissions with respect to the NACK-only feedback scheme because the gNB knows the set of UEs that have failed to decode previous TXs. However, ACK/NACK feedback requires higher UL resource consumption, which scales linearly with the number of UEs in the multicast group. In Section 4, we use system-level simulations to study the influence of the used feedback scheme on the overall resource consumption in different scenarios and provide recommendations for selecting the feedback scheme for mURLLC traffic.

### 3.3. Sounding Period

The choice of short-term transmission parameters, such as precoder and MCS (see the following sections for details), significantly depends on the accuracy of channel measurements available at the gNB. In the case of TDD, the gNB measures both DL and UL channels based on SRS signals periodically transmitted by each UE in UL slots. As we show in this paper, the choice of SRS period can significantly influence the overall channel resource consumption for a multicast stream. In particular, a low SRS period improves the accuracy of channel measurements and, therefore, reduces the DL resource consumption. However, it significantly increases UL resource consumption used for pilot signals, which scales with the number of UEs in the multicast group. In contrast, a high SRS period reduces sounding overhead but increases DL resource consumption because of selecting too-low MCSs. In Section 4, we consider scenarios with different UE mobility and study how to select the SRS period in order to find a good balance between DL resources consumed for data transmission and UL resources consumed for sounding.

### 3.4. Precoder Selection

For each transmission, the gNB constructs a special matrix called a precoder that determines how signals are generated from different gNB antennas. A proper precoder can significantly boost the performance of the M-MIMO system. The corresponding problem for one-antenna UEs is formulated as follows. For each UE *i* and RB *j*, the gNB has estimations of (i) the channel matrix $H_{i,j}$ of size 1×M that is updated based on periodic SRS, and (ii) the interference plus noise power $\sigma_{j}^{2}$ in RB *j*, which is obtained based on the channel quality indicator reports provided by the UEs. For each RB *j* used for the DL transmission, the gNB constructs a precoder $W_{j}$, which is a matrix of size M×1. The Signal to Interference plus Noise Ratio (SINR) for UE *i* in RB *j* is estimated as:

(1) $$SINR_{i,j}\left(W_{j}\right)=\frac{|H_{i,j}W_{j}{|}^{2}}{\sigma_{j}^{2}}.$$

Since the UE with the worst SINR limits multicast transmission parameters, the precoder selection problem for the set *U* of UEs is stated as follows:

(2) $$\begin{array}{c} \begin{array}{cc} \; & \max_{W_{j}}\left[\min_{i\in U}SINR_{i,j}\left(W_{j}\right)\right],\\ \; & s.t.:\;{∥W_{j}∥}_{F}^{2}\leq P_{TX}, \end{array} \end{array}$$

where $P_{TX}$ is the transmission power allocated for a single RB. In the literature, authors often consider a similar problem statement [37]:

(3) $$\begin{array}{c} \begin{array}{cc} \; & \min_{W_{j}}{∥W_{j}∥}_{F}^{2},\\ \; & s.t.:\;SINR_{i,j}\left(W_{j}\right)\geq \gamma ,\;\forall i\in U, \end{array} \end{array}$$

where γ is the SINR constraint. Problems (2) and (3) are similar in the sense that the solution to Problem (2) gives the solution to Problem (3) with a proper scaling, which depends on constraints $P_{TX}$ and γ. Both optimization problems are non-convex and proven to be NP-hard in case M≤N [37].

Let us classify and analyze numerous approaches to the multicast precoding problem. One of the approaches is to reformulate Problem (3) as follows (see [37]):

(4a) $$\begin{array}{cc} \; & \min_{X_{j}}trace\left(X_{j}\right), \end{array}$$

(4b) $$\begin{array}{cc} \; & s.t.:\;trace\left(X_{j}Q_{i,j}\right)\geq \gamma \sigma_{j}^{2},\;\forall i\in U, \end{array}$$

(4c) $$\begin{array}{cc} \; & X_{j}⪰0, \end{array}$$

(4d) $$\begin{array}{cc} \; & rank(X_{j})=1, \end{array}$$

where $X_{j}=W_{j}W_{j}^{H}$, $Q_{i,j}=H_{i,j}^{H}H_{i,j}$, trace(X) and rank(X) denote the trace and rank of matrix *X*, respectively. Note that the objective function (4a) and constraints (4b) and (4c) are convex, while only the constraint (4d) is non-convex. By relaxing the constraint (4d), we obtain the Semi-Definite Relaxation (SDR) (4a), (4b), (4c) of the optimization problem, Problem (4). That SDR can be solved with state-of-the-art convex solvers. However, the obtained SDR solution gives the solution to Problems (3) and (4) only if the matrix $X_{j}$ has rank equal to one. Otherwise, it only provides a lower bound on the objective function for Problem (3) and a corresponding upper bound for the Problem (2). Thus, we use that upper-bound solution to evaluate the performance of other precoder algorithms.

Since the usage of convex solvers results in high computational complexity inappropriate for mURLLC applications with tight latency requirements, below we consider different approaches proposed in the literature that have lower computational complexity.

Mohammadi et al. [35] propose the application of the Successive Convex Approximations (SCA) method to the Problem (3) and use the Alternating Direction Method of Multipliers (ADMM) on each SCA iteration, which is a relatively low-complexity state-of-the-art method for convex problems. Unfortunately, the resulting algorithm, which we refer to as SCA-ADMM, still has high complexity that depends on the convergence threshold as we show in Section 4.

Other approaches considered in the literature are based on different heuristics. For example, Hunger at al. [33] derived a closed-form solution for multicast Problem (3) with only two UEs, and presented an FF-C2 (Full Featured Combine-2) algorithm that performs a full search over all possible pairs of UEs. They also proposed a heuristic to reduce the search space, which is called an RC-C2 (Reduced Complexity Combine-2) algorithm.

In Silva and Klein [31], the authors consider adaptations of well-known unicast beamforming algorithms to the multicast case, e.g., multicast versions of Maximum Ratio Transmission (mMRT) [19,32], Zero Forcing (mZF), and Minimum Mean Square Error (mMMSE) precoders. Since these algorithms require relatively simple linear algebra operations, they have very low computational complexity; however, they have very poor performance as we show in Section 4.

The trade-off between computational complexity and performance can be reached by iterative algorithms that use relatively simple (compared to SCA-ADMM) linear algebra operations on each iteration. Examples are Iterative Update (IU) [33] and Multiplicative Update (MU) [20] algorithms that on each iteration increase the objective function value for Problem (2) (in case of IU algorithm) or, in case of the MU algorithm, for the following proportional fair problem:

(5) $$\begin{array}{c} \begin{array}{cc} \; & \max_{W_{j}}\left[\frac{1}{2}\sum_{i\in U}\log\left(SINR_{i,j}\left(W_{j}\right)+\zeta \right)\right],\\ \; & s.t.:\;{∥W_{j}∥}_{F}^{2}\leq P_{TX}, \end{array} \end{array}$$

where ζ is a small constant that is necessary for the numerical stability of the MU algorithm.

Finally, such algorithms as SBFC (Successive Beamforming-Filter Computation) [33] or QR decomposition-based algorithm, called, hereafter, the QR [34] construct precoder, as a linear combination of orthogonal vectors that form a linear subspace of UE channels. Each vector and corresponding coefficient in a linear combination are selected to increase the SINR of each particular UE, thus satisfying their constraint in optimization problem, Problem (3).

We study the above-described precoder construction algorithms in Section 4.2 and select those that best provide low complexity and high performance with respect to the SDR-based upper bound. Note that the channel matrix $H_{i,j}$ may significantly change with time, while the precoder is constructed based on its periodic SRS measurements. Thus, we also study the influence of the SRS period on the performance of the selected precoders.

Let us now consider how the gNB selects the precoder for different transmission attempts. For the first TX, the gNB constructs a precoder for the set *U* that contains all recipients of the multicast stream. The target set for the second TX depends on the used feedback scheme. In the case of NACK-only feedback, the gNB does not know which UEs have failed to decode a packet. Thus, the gNB uses the same set *U* as for the first TX. In the case of ACK/NACK feedback, the gNB knows the exact set $U_{f}$ of UEs that have failed to decode the first TX. Having the lower number of UEs in the set $U_{f}$, the gNB can change the precoder and increase SINR for these UEs in the second TX.

### 3.5. RB Allocation

For each slot, the gNB determines which RBs are used for transmission of enqueued multicast packets. The RB allocation procedure consists of two steps. First, the gNB divides RBs between various multicast streams (packets). Second, it allocates particular RBs to each multicast packet.

Khorov et al. [28] evaluate various scheduling policies for unicast URLLC traffic. They show that the well-known Earliest Deadline First (EDF) policy provides high network capacity for URLLC and has very low complexity. This policy can be adapted to mURLLC traffic as follows. Let P={1, …, P} be the set of multicast packets pending transmission and B={1, …, B} be the set of available RBs. First, the gNB sorts enqueued multicast packets in the ascending order of their remaining lifetimes: $RT_{p}=D^{QoS}-D_{p}$, where $D_{p}$ is the packet *p* queuing delay. Note that packets with $D_{p}>D^{QoS}$ are dropped. Having sorted a set of packets $\hat{P}$, the gNB considers the first packet and allocates RBs to this packet as described in the following paragraph until all its bytes are transmitted or no free RBs are available. Then the gNB considers the second packet in $\hat{P}$ and so on.

Two approaches can be used to allocate particular RBs to packets: Frequency-Selective (FS) and non-FS scheduling. With FS scheduling, the gNB takes into account the channel quality in the considered RBs and allocates the best RBs to packets in order to minimize resource consumption. For multicast traffic, the FS scheduling is implemented as follows. For each RB j∈B, the gNB determines the recipient with the worst SINR, i.e., $SINR_{j,p}=\min_{i\in U_{p}}SINR_{i,j}$, where $U_{p}$ is the set of recipients for packet *p* and $SINR_{i,j}$ is SINR of UE *i* in RB *j* given by (1). Then, the gNB sorts free RBs in set B in the descending order of $SINR_{j,p}$ and allocates free RBs until all bytes of the considered packet are transmitted or no free RBs are available. With non-FS scheduling, the gNB assumes that RBs have the same quality. Thus, it can select RBs sequentially or randomly.

FS and non-FS scheduling approaches both have their benefits and drawbacks. In terms of performance, FS scheduling provides lower resource consumption and, thus, increases the network capacity. However, in terms of complexity, FS scheduling requires the calculation of the precoder in each RB (i.e., to estimate $SINR_{j,p}$). In contrast, with non-FS scheduling, the gNB only needs to calculate the precoder in the allocated RBs. In Section 4, we study in detail this tradeoff between performance and complexity.

### 3.6. MCS Selection

For each transmission attempt and selected RBs, the gNB shall determine a single MCS. In particular, for a transmission attempt *t*, the gNB shall find the highest MCS $MCS_{t}$ that provides a block error rate (BLER) for a multicast group below the target value $p_{t}$. The MCS selection procedure consists of two steps. First, the gNB uses the error model to find the highest MCS $MCS_{SINR}$ that provides BLER below $p_{t}$ for a given set of worst SINRs (i.e., $SINR_{j}$ in the allocated RBs). Since the wireless channel may significantly change with time, the precoder and SINR estimations quickly become outdated, and $MCS_{SINR}$ may not provide the required reliability. To address this issue, at the second step, the gNB adjusts the MCS as discussed in detail below.

#### 3.6.1. One TX Case

The method to adjust the MCS depends on the used feedback scheme. For No feedback, the gNB does not have information about actual BLER at the receivers. Thus, to provide high reliability, the gNB selects a robust MCS to take into account possible channel fluctuations. In particular, the authors of [30] propose a simple method (called eOLLA) that subtracts a positive constant Δ(N) from $MCS_{SINR}$ to take into account possible degradation of SINR at UEs: $MCS_{1}=MCS_{SINR}-\Delta \left(N\right)$. The exact value of Δ(N) is selected based on long-term experiments as the value providing the required reliability for a given multicast group size *N*.

For the schemes with UE feedback, the MCS can be dynamically adjusted using an Outer Loop Link Adaptation (OLLA) algorithm [36]. While OLLA is a widely used algorithm for unicast, below we propose its multicast version.

With multicast OLLA, the gNB keeps a single subtraction $\Delta_{olla}$ for a multicast group, and the MCS is selected as $MCS_{1}=MCS_{SINR}-round\left(\Delta_{olla}\right)$. The gNB updates $\Delta_{olla}$ based on the obtained HARQ feedback. Specifically, $\Delta_{olla}$ is increased by a constant $\delta_{+}$ if the transmission fails as defined at the beginning of Section 2. Otherwise, it is reduced by a constant $\delta_{-}$. The average BLER provided by the multicast OLLA algorithm converges to $p_{olla}=\frac{\delta_{-}}{\delta_{-}+\delta_{+}}$. Therefore, in the One TX case, we can set $p_{1}=PLR^{QoS}$ and accordingly select OLLA parameters.

#### 3.6.2. Two TXs Case

In this case, the gNB selects two MCSs. The simplest approach considered in many papers is to use the same MCS for the first and the second transmissions: $MCS_{2}=MCS_{1}$, where $MCS_{1}$ is selected using the OLLA algorithm with the target BLER $p_{1}=\sqrt{PLR^{QoS}}$. By taking into account HARQ combining gain, it is assumed that $p_{2}\leq p_{1}$ and, thus, the overall reliability requirement is satisfied.

Since 3GPP specifications allow the use of different MCSs for various transmission attempts, we propose the following approach. We select two target BLERs such that $p_{1}\cdot p_{2}\leq PLR^{QoS}$ (the specific configurations are analyzed in Section 4). For each TX, the target BLER is provided by a separate OLLA adjustment. Note that in the case of ACK/NACK feedback, SINRs for the second TX might be higher than for the initial transmission and, thus, selecting $MCS_{2}>MCS_{1}$ reduces resource consumption for retransmissions.

## 4. Performance Evaluation

### 4.1. Simulation Setup

To evaluate the performance of the algorithms presented in Section 3, we have significantly extended the system-level simulator NS-3 [38] by implementing the new multicast mechanisms introduced in 3GPP specifications, M-MIMO features, and multicast traffic.

Unless otherwise explicitly stated, we consider an Urban Macro scenario with *N* UEs randomly distributed in the gNB coverage area. Both LOS and NLOS channels are modeled. UEs move with a 3 kmph speed and send SRS with a 20 ms period. The gNB uses the FS EDF scheduler described in Section 3.5. Table 3 lists the main simulation parameters.

In the experiments, we measure (i) the average PLR, and (ii) the average DL and UL resource consumption. The lower the resource consumption, the higher the mURLLC capacity. The DL resource consumption is determined as the average number of RBs used in a DL slot for data transmission divided by the total number of RBs. The UL resource consumption consists of two parts. First, some UL resource is used for UEs’ feedback transmission. For the ACK/NACK scheme, the resource consumption can be found as $\eta_{f}=\frac{NB_{f}}{B(k_{dl}+k_{ul})}$, where $B_{f}$ is the number of RBs allocated for transmission of a single UE feedback (by default, $B_{f}=4$ RBs). For the NACK-only scheme, the resource consumption is *N* times lower because a single resource is allocated for all UEs. Second, UL resource is used for SRS transmission: $\eta_{s}=\frac{NT_{slot}}{k_{0}N_{0}T_{SRS}}$, where $k_{0}=14$ is the number of OFDM symbols in a slot, and $N_{0}=2$ is the number of SRSs that is multiplexed in an OFDM symbol.

In the following sections, we analyze the performance of the various transmission parameter selection algorithms described in Section 3. To simplify the evaluation, we changed the order compared with Section 3. Specifically, since the literature provides dozens of precoder selection algorithms that significantly affect performance, we start our analysis with their comparison. Based on the analysis, we determine the best precoder selection algorithms for mURLLC. After that, we evaluate other short-term transmission parameters selection algorithms (i.e., RB and MCS selection). Since the implementation of the MCS selection algorithm depends on the maximum number of TX attempts, we evaluate the joint effect of MCS and the number of TX attempts selection. Finally, we study the influence of long-term parameters (the feedback scheme and the SRS period) on the overall system performance.

### 4.2. Analysis of Precoder Selection Algorithms

**Figure 2:**
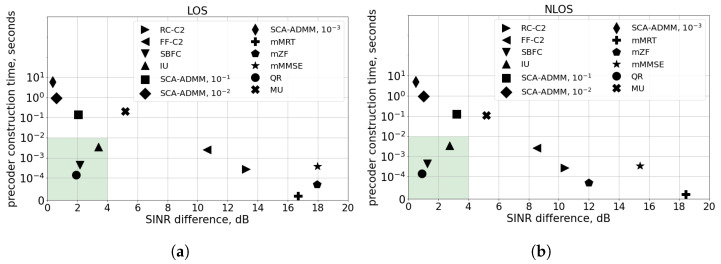
Scenario with N=20 UEs, M=64 antennas at gNB, 3 kmph mobility, and $T_{SRS}=20$ ms: (**a**) LOS channel, (**b**) NLOS channel.

**Figure 3:**
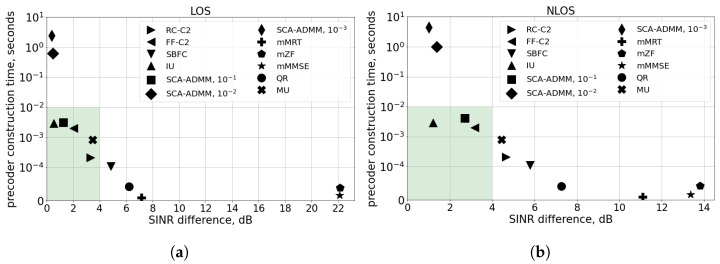
Scenario with N=20 UEs, M=4 antennas at gNB, 3 kmph mobility, and $T_{SRS}=20$ ms: (**a**) LOS channel, (**b**) NLOS channel.

Let us start with the performance comparison of precoder selection algorithms. For this, we sampled the values of the channel matrix $H_{i,j}$ in all RBs for 20 UEs using the NS-3 channel model. For each matrix, we construct the precoder with algorithms from Section 3.4. The X-axis in Figure 2 and Figure 3 corresponds to the average difference between the minimal SINR $SINR_{j}$ obtained with the considered algorithm and the upper bound obtained with the SDR approach. The Y-axis is the mean time needed to construct a single precoder with a 3.3 GHz Intel Core i3-2120 processor [39]. Note that the gNB can implement two approaches to compute the precoder: (i) offline, in which the precoder is calculated in advance for each multicast group when the gNB receives the corresponding SRSs, and (ii) online, in which the precoder is calculated each time the gNB schedules transmission to a particular multicast group. For the offline approach, the precoder computation time should be much lower than $T_{SRR}$, while for the online approach, the precoder computation time should be much lower than $D^{QoS}$. Since in our experiments, $T_{SRR}$ = 20 ms and $D^{QoS}$ = 10 ms, we consider 10 ms as the reference value which limits the precoder computation time. If the precoder construction algorithm has a computational time greater than 10 ms, we consider it unacceptable for mURLLC applications.

Figure 2 shows the results for the M-MIMO case (i.e., 64 antennas at the gNB) for both LOS and NLOS channel models. Let us start with an analysis of the SCA-ADMM algorithm. For this algorithm [35], we check different convergence thresholds ε. We can see that the SINR difference for the SCA-ADMM algorithm reduces for a stricter convergence threshold ε. However, even for the highest convergence threshold $\varepsilon =10^{-1}$, the corresponding precoder construction time is ≈0.1 s, which is not acceptable for mURLLC applications. The FF-C2 and RC-C2 algorithms [33], which search a multicast precoder over UE pairs, provide reasonable construction time 1−10 ms. However, as each precoder in a search space takes into account only two considered UEs, their SINR is much lower than the SDR upper bound. Multicast adaptations of linear beamforming algorithms from [31], such as mMRT, mZF, and mMMSE, provide the lowest precoder construction time. However, they provide the worst SINRs among considered solutions. The IU and MU algorithms, which iteratively construct precoders using simple linear algebra operations, show much better performance. As mentioned in Section 3.4, the MU algorithm optimizes the proportional fair objective function from Problem (5) instead of the max–min SINR objective function from Problem (2). Thus, the IU algorithm provides better performance than the MU algorithm in terms of minimal SINR over RBs. Finally, the SBFC and QR algorithms, which construct the precoder as a linear combination of UE channels, provide the SINR closest to the upper bound with reasonable construction time.

Let us analyze how the number of antennas influences precoder construction algorithm performance and complexity. For that, in Figure 3, we consider the case of four antennas at the gNB, corresponding to 4G systems. Interestingly, the performance of the QR and SBFC algorithms (which are the best in the M-MIMO case) significantly degrade for a low number of antennas because they use orthogonal projections of UE channels on the precoder null-space. This procedure significantly reduces SINR because the UE channels become non-orthogonal when the number of antennas reduces. In contrast, the IU and MU algorithms do not use this orthogonalization procedure and show much better performance. Multicast adaptations of linear beamforming algorithms (mMRT, mZF, and mMMSE) provide the lowest computation time but the worst performance, which is explained as follows. In the case of a low number of antennas, the mZF algorithm requires the computation of the pseudoinverse of an underdetermined matrix, while the mMMSE algorithm requires the inversion of an ill-determined matrix. Because of the lower number of antennas, the complexity of the SCA-ADMM algorithm with convergence threshold $\varepsilon =10^{-1}$ significantly reduces. Thus, it can be considered as the candidate solution.

From the two considered above cases, we can see that the performance and complexity of various precoders significantly depends on the number of antennas: for 4G systems with low number of antennas, IU, SCA-ADMM $\varepsilon =10^{-1}$, and FF-C2 provide a good balance between performance and complexity, while, for 5G M-MIMO systems, QR, SBFC, and IU show better results. Thus, a particular algorithm shall be selected taking into account the configuration of antennas and the complexity constraints at the gNB. For that, a preliminary link-level evolution similar to that presented in Figure 2 and Figure 3 can be carried out based on the real channel measurements.

**Figure 4:**
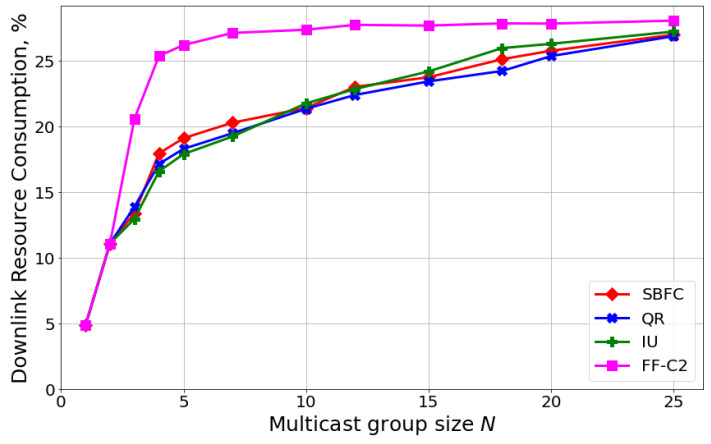
Scenario with 3 kmph mobility, a single TX, the NACK-only feedback scheme, $p_{1}=PLR^{QoS}$, and $T_{SRS}=20$ ms.

Let us analyze the performance of the best precoder selection algorithms (SBFC, QR, IU, and FF-C2) using system-level simulations that take into account the channel aging effect, RB allocation, and MCS selection algorithms. In particular, in Figure 4, we consider 5G M-MIMO systems with 64 antennas, the One TX scheme with NACK-only feedback, and MCS is selected based on OLLA with $p_{1}=PLR^{QoS}$. We can see that the conclusions for 20 UEs coincide with those of Figure 2: the performances of the SBFC, IU, and QR precoders are close to each other (the difference is below 5%), while FF-C2 provides higher DL resource consumption because of lower SINR. So, proper configuration of the precoder algorithm can reduce resource consumption by more than 25% and allows the precoder to be computed in real time (i.e., precoder construction time is comparable to $D^{QoS}$).

### 4.3. Analysis of RB Allocation Algorithms

**Figure 5:**
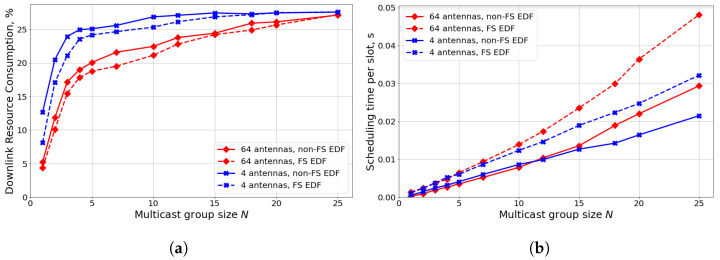
Scenario with 3 kmph mobility and $T_{SRS}=20$ ms: (**a**) downlink resource consumption, (**b**) scheduler execution time.

Let us analyze the performance of FS and non-FS scheduling approaches. Similar to the previous section, we consider different numbers of antennas at the gNB: (i) 4 antennas, corresponding to 4G systems, and (ii) 64 antennas, corresponding to 5G M-MIMO systems. Figure 5a shows the following results. First, a higher number of antennas significantly increases SINR at UEs and reduces the DL resource consumption (from 20 to 40% depending on the number of UEs in the multicast group). Second, the usage of frequency selectivity (i.e., FS scheduling) provides lower resource consumption compared to non-FS scheduling. However, the gain of FS scheduling depends on the number of UEs and the number of antennas. Specifically, for a single UE, FS scheduling reduces resource consumption by 35% for 4 antennas and only by 15% for 64 antennas. The lower gain in the case of M-MIMO is explained by the channel hardening effect [40]: a higher number of antennas reduces the channel quality fluctuation both in time and frequency domains. When the number of UEs increases, the gain of FS scheduling significantly reduces. Specifically, when the multicast group includes more than ten UEs, the difference between FS and non-FS scheduling is less than 5% for both considered antenna configurations. The reason is that the channel quality in the RB *j* is determined by the UE with the lowest SINR: $SINR_{j}$. For a higher number of UEs, the difference between $SINR_{j}$ in different RBs reduces.

Figure 5b shows the average time needed to compute the schedule (including precoder construction) for a single slot. The results show that non-FS scheduling allows a significant reduction in the scheduler complexity—by 30% for 4 antennas and by 40% for 64 antennas. So, we can conclude that FS scheduling is fruitful only when the multicast group consists of a few UEs and the gNB has few antennas. For large multicast group size and M-MIMO systems, non-FS and FS scheduling approaches provide almost the same performance but the former has much lower computational complexity.

### 4.4. Influence of the Maximum Number of Transmission Attempts

**Figure 6:**
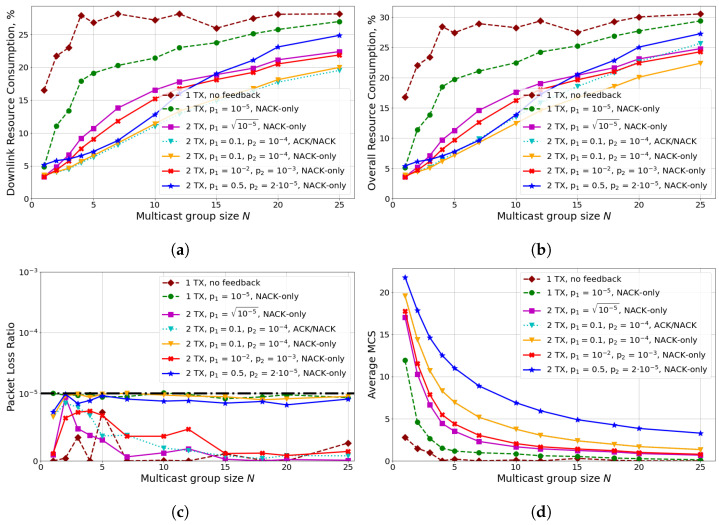
Scenario with 3 kmph mobility and $T_{SRS}=20$ ms: (**a**) downlink resource consumption, (**b**) overall resource consumption, (**c**) packet loss ratio, (**d**) average MCS.

Let us consider a 5G M-MIMO system with 64 antennas at the gNB. The precoder construction algorithm is SBFC. Figure 6 shows the results for various configurations of transmission parameters (i.e., the number of transmission attempts, the MCS selection algorithm, and the feedback scheme).

First, we can see that for all the considered configurations, strict URLLC reliability and latency requirements are satisfied. Specifically, according to Figure 6c, PLR is below $PLR^{QoS}$. Note that the packet is assumed lost if it is not delivered within $D^{QoS}$. Thus, the latency requirement is also satisfied.

Second, let us analyze the influence of the number of transmission attempts. The main observation from the obtained results is that because of strict reliability requirements, with a single TX, the gNB uses too-low a MCS (see Figure 6d), which increases resource consumption too much. Though for a single TX, NACK-only feedback does not induce retransmissions, it allows dynamic MCS adjustment with the OLLA algorithm, which reduces resource consumption by up to 70% compared to the No feedback scheme with eOLLA MCS selection algorithm (see Section 3.6). Note that the curve ‘1 TX no feedback’ is non-monotonic and non-smooth because eOLLA uses a discrete set of MCS adjustments Δ(N). Thus, MCS adjustment changes significantly when the number of UEs changes. In contrast, for other curves corresponding to the OLLA algorithm, the MCS adjustment $\Delta_{olla}$ is a real number, which changes smoothly. Switching from one TX to two TXs (i.e., the usage of multicast HARQ) reduces resource consumption up to three times. Note that this effect differs from that observed for loss-tolerant traffic (e.g., IPTV) [30], where multicast HARQs only negligibly reduce resource consumption compared with a single TX. So, in the case of URLLC with strict latency and reliability requirements, if the latency budget allows, the gNB shall enable multicast HARQ to reduce resource consumption.

### 4.5. Analysis of MCS Selection Algorithms

Let us now consider the influence of the MCS selection algorithm and its parameters (i.e., target BLERs). The main observation is that for the two TXs cases, different target BLERs for the first TX and the second TX may significantly reduce channel resource consumption. Specifically, the usage of target BLERs $p_{1}=0.1,p_{2}=10^{-4}$ reduces DL resource consumption by up to 40% compared to the case with $p_{1}=p_{2}=10^{-2.5}$ because for the first TX, providing BLER of the order of $10^{-2.5}$ requires much more resources than 0.1. The resources for the second TX are consumed only when the first TX fails with the probability $p_{1}$. This effect significantly differs from the one observed for delay-tolerant broadband traffic for which all TXs are carried ou with the same target BLERs and MCSs. To further elaborate on that observation, we consider the downlink resource consumption of only the first TX $\theta_{1}\left(p_{1}\right)$ and total consumption of the first TX and second TX (if any) $\theta_{2TX}\left(p_{1}\right)$. As we select $p_{1}\cdot p_{2}=PLR^{QoS}$, downlink resource consumption for second TX can be estimated as $\theta_{1}\left(\frac{PLR^{QoS}}{p_{1}}\right)$, and $\theta_{2TX}\left(p_{1}\right)$ is:

$$\theta_{2TX}\left(p_{1}\right)=\theta_{1}\left(p_{1}\right)+p_{1}\cdot \theta_{1}\left(\frac{PLR^{QoS}}{p_{1}}\right),$$

**Figure 7:**
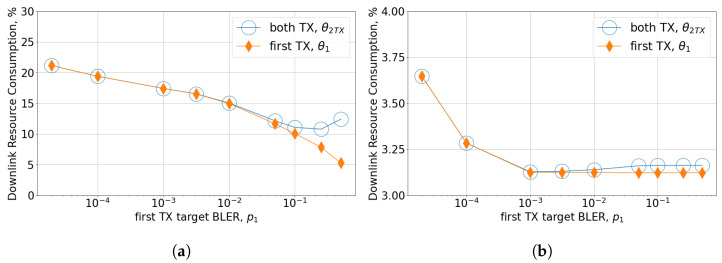
Scenario with N=10 UEs, 3 kmph mobility, and $T_{SRS}=20$ ms: (**a**) NLOS, (**b**) LOS.

Figure 7 shows $\theta_{1}\left(p_{1}\right)$ and $\theta_{2TX}\left(p_{1}\right)$ for the NLOS and LOS scenarios. In the NLOS scenario, $\theta_{1}$ decreases monotonically with $p_{1}$, and the minimum of $\theta_{2TX}$ is achieved at relatively high $p_{1}=0.25$. In contrast, in the LOS scenario, $\theta_{1}$ achieves a plateau after $p_{1}=10^{-3}$, since even the highest possible MCS satisfies target BLER $10^{-3}$. Because of this, the minimum of $\theta_{2TX}$ in the LOS scenario is achieved at $p_{1}=10^{-3}$. Note that the target BLER $p_{1}=0.1$, which is widely used by default for broadband traffic, provides downlink resource consumption close to that of the minimum (the difference does not exceed 10%). Thus, the selection of $p_{1}∼0.1$ recommended for broadband traffic provides sub-optimal results for both considered scenarios. However, in contrast to broadband traffic, the target BLER for the second TX should be selected to guarantee high reliability. Further, we consider the MCS selection scheme with two target BLERs $p_{1}=0.1$ and $p_{2}=10^{-4}$.

### 4.6. Comparison of Feedback Schemes

Let us compare the performance of various feedback schemes. ACK/NACK feedback tells gNB which UEs failed to receive the first TX. As the average number of intended receivers for the second TX is much lower than for the first one, it allows increasing SINR, selecting higher MCS for the second TX, and reducing the DL channel resource consumption. However, the comparison between ACK/NACK and NACK-only feedbacks (see Figure 6a) shows that the gain is tiny. Moreover, as ACK/NACK feedback consumes too many UL resources, the overall gain of the ACK/NACK scheme is negative as shown in Figure 6b. Thus, the main conclusion is that NACK-only feedback provides a good balance between DL and UL resource consumption for mURLLC.

### 4.7. Influence of the UE Mobility and Sounding Period

**Figure 8:**
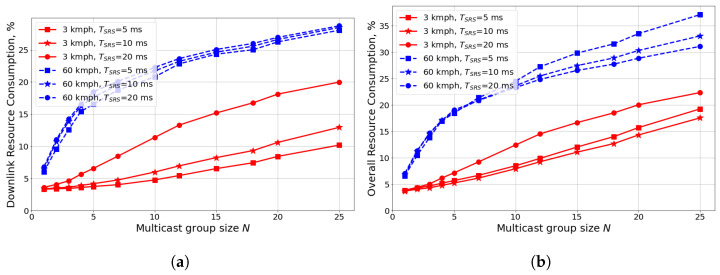
Scenario with different $T_{SRS}$ and UE mobility: (**a**) downlink resource consumption, (**b**) overall resource consumption.

As mentioned in Section 3.4, the channel matrix may significantly change with time while the precoder is constructed based on its periodic SRS measurements. This problem is known as precoder aging. In Figure 8, we study the influence of UE mobility and $T_{SRS}$ on the performance of the best configuration of transmission parameters obtained in the previous sections: 2 TXs, $p_{1}=0.1$, $p_{2}=10^{-4}$, and NACK-only feedback.

We see that small $T_{SRS}$ reduces the DL resource consumption for the 3 kmph case by up to 60% because of more frequent channel estimation. However, for 60 kmph, the gain is below 10% because the channel information quickly becomes outdated. At the same time, because of the large number of receiving UEs per stream, the amount of UL channel resources required for SRS is of the same order as for data transmission. Consequently, considering the overall resource consumption (see Figure 8b) for low mobility, the gain of selecting optimal $T_{SRS}$ diminishes, and the optimal value of $T_{SRS}$ changes. For high mobility, $T_{SRS}=5$ ms—being the best option for DL resource consumption—is the worst one for the overall resource consumption. Thus, the main observation is that because of a large number of receiving UEs and typically low traffic intensity, mURLLC induces so much channel sounding overhead per stream that obtaining frequent channel information becomes inefficient. To reduce SRS overhead, new adaptive sounding and UE clusterization schemes should be developed that make UEs send SRSs with different periods based on their locations. For example, we can select low $T_{SRS}$ for cell-edge UEs with low SINRs, while selecting high $T_{SRS}$ for cell-center UEs.

## 5. Conclusions

In this paper, we studied the new mechanisms introduced in 3GPP specifications that enabled multicast in 5G systems. We analyzed how to efficiently configure these mechanisms and how to adapt transmission parameter selection algorithms (i.e., precoder selection, RB allocation, and MCS selection) to provide reliable delivery of an mURLLC stream with low channel resource consumption. Based on the extensive simulation results, we provide the following recommendations (see Table 2 for details).

1. The performance and complexity of various precoder selection algorithms significantly depend on the number of antennas at the gNB. For the M-MIMO case, orthogonal subspace construction algorithms (e.g., SBFC, QR) provide the lowest resource consumption with low complexity;
2. The usage of the FS EDF scheduler notably reduces the channel resource consumption only for low multicast group size and a low number of antennas at the gNB. In other cases, the non-FS EDF scheduler provides almost the same resource consumption and up to 40% lower computational complexity;
3. If the latency budget allows HARQ retransmissions, they shall be enabled because, in contrast to traditional broadband multicast traffic, they allow reducing resource consumption up to three times for mURLLC;
4. In the case of two transmissions, the usage of two different target BLERs for MCS selection significantly reduces resource consumption compared with the widely used approach of selecting the same MCS for the initial transmission and retransmission;
5. Out of three considered feedback schemes, the NACK-only scheme provides the lowest resource consumption for mURLLC;
6. In the case of mURLLC, optimization of the sounding period allows a notable reduction in resource consumption only in low-mobility scenarios.

Summing up, by implementing the recommendations above, the network operator can provide mURLLC service with much lower channel resource consumption compared with the baseline solutions proposed for broadband multicast or unicast URLLC traffic and, therefore, significantly increase the network capacity in terms of the number of concurrent mURLLC flows or their aggregated load.

One of the promising directions for future research is to adaptively select the best sounding period for each multicast group member.

## Figures and Tables

**Table 1:** Inheritance of Multicast URLLC Solutions from Multicast eMBB and Unicast URLLC Solutions.

Problem	Multicast eMBB		Multicast URLLC		Unicast URLLC
The maximum number of transmission attempts (TXs)	One TX attempt		Two TX attempts (if the delay budget allows, use HARQ retransmissions)	⇐	Two TX attempts (if the delay budget allows, use HARQ retransmissions)
Feedback Scheme	No feedback	X	NACK-only feedback	X	ACK/NACK feedback
Sounding period	The optimal period is scenario-dependent	X	Optimization of the sounding period is fruitful only for low-mobility scenarios	X	The optimal period is scenario-dependent
Precoder selection	QR, SBFC, IU, MU, SCA-ADMM, FF-C2,RC-C2.	⇒	QR, SBFC, IU		MRT, ZF, MMSE
Scheduler	FS PF and its modifications.		Multicast adaptation of (non-)FS EDF	$\overset{new}{⇐}$	FS EDF
MCS selection	Fixed subtraction (eOLLA)		Multicast adaptation of OLLA with two target BLERs	$\overset{new}{⇐}$	OLLA

**Table 2:** Summary of the considered problems and possible solutions.

Time Scale	Problem	Possible Solutions	Recommendations
Long-term parameters	The maximum number of transmission attempts (TXs)	For mURLLC requirements, typically one or two transmission attempts are possible (see Section 3.1).	If the latency budget allows HARQ retransmissions, they shall be enabled. They reduce resource consumption up to three times compared with a single transmission attempt (see Section 4.4).
	Feedback Scheme	Three schemes are described in 3GPP specifications (see Section 3.2): No feedback; ACK/NACK feedback; NACK-only feedback.	Out of the three schemes, the NACK-only feedback provides the lowest overall (DL+UL) resource consumption (see Section 4.6).
	Sounding period	The sounding period can be tuned by the gNB. A higher sounding period improves the quality of the precoder (i.e., increases SINRs at UEs) but increases UL overhead (see Section 3.3).	For low UE speed, sounding period optimization can reduce resource consumption up to 40%. For high UE speed, a high sounding period only increases UL overhead without a notable reduction of DL resource consumption (see Section 4.7).
Short-term parameters (for each TX)	Precoder	Existing algorithms can be classified into (see Section 3.4): Heuristics based on unicast case (mMF, mZF, mMMSE) [19,31,32]; Other heuristics (FF-C2, RC-C2) [33]; Iterative algorithms (IU, MU) [20,33]; Orthogonal subspace construction (SBFC, QR) [33,34]; Convex optimization (SCA-ADMM) [35].	For a low number of antennas, iterative algorithms provide the highest performance with reasonable complexity. For the M-MIMO case, orthogonal subspace construction algorithms provide better results (see Section 4.2).
	RB allocation (scheduler)	The EDF scheduler aims to minimize the latency required for mURLLC [28]. It can be implemented in several ways (see Section 3.5): Frequency-Selective (FS); Non-Frequency-Selective (non-FS).	FS EDF provides notable gain only for a low number of UEs and a low number of antennas at the gNB. In other cases, Non-FS EDF provides the same performance with up to 40% lower complexity (see Section 4.3).
	MCS selection	MCS selection depends on the feedback scheme (see Section 3.6). For no feedback: eOLLA solution [30]; For ACK/NACK or NACK-only feedback: multicast adaptation of OLLA [36] with different target BLERs (MCSs) in case of two TXs.	The usage of feedback and multicast OLLA up to three times reduces the overall resource consumption. In the case of two TXs, different target BLERs for the first and second TXs reduce resource consumption up to 40% compared with the same BLERs (see Section 4.5).

**Table 3:** Main simulation parameters.

Parameter	Value
Carrier frequency	3.6 GHz
Bandwidth	100 MHz
TDD structure	$k_{dl}=8$, $k_{ul}=2$, $T_{slot}=0.5$ ms
Number of RBs	256
Channel model	3GPP TR 38.901, Urban Macro
gNB/UE antennas	64 or 4
UE antennas	1
gNB/UE TX power	33/23 dBm
Traffic	CBR: 400 bytes, 1 ms period
QoS requirements	$PLR^{QoS}=10^{-5}$, $D^{QoS}=10$ ms
Simulation time	1000 s, 40 runs

## Abbreviations

The following abbreviations are used in this manuscript:

3GPP	3rd Generation Partnership Project
ACK	Acknowledgment
ADMM	Alternating Direction Method of Multipliers
DL	Downlink
FF-C2	Full Featured Combine-2
HARQ	Hybrid Automatic Repeat Request
IU	Iterative Update
gNB	next generation NodeB
MCS	Modulation and Coding Scheme
M-MIMO	Massive Multiple-Input, Multiple-Output
mMMSE	multicast Minimum Mean Square Error
mMRT	multicast Maximum Ratio Transmission
MU	Multiplicative Update
mURLLC	multicast Ultra-Reliable Low-Latency Communications
mZF	multicast Zero Forcing
NACK	Negative Acknowledgment
PLR	Packet Loss Ratio
QoS	Quality of Service
RB	Resource Block
RC-C2	Reduced Complexity Combine-2
SBFC	Successive Beamforming-Filter Computation
SCA	Successive Convex Approximations
SDR	Semi-Definite Relaxation
SINR	Signal to Interference plus Noise Ratio
SRS	Sounding Reference Signal
TDD	Time Division Duplex
TX	Transmission attempt
UE	User Equipment
UL	Uplink
URLLC	Ultra-Reliable Low-Latency Communications
